# Urban–Rural Disparities in Non-Adherence to Iron Supplementation Among Pregnant Women Aged 15 to 49 in Sub-Saharan Africa

**DOI:** 10.3390/ijerph22060964

**Published:** 2025-06-19

**Authors:** Yibeltal Bekele, Bircan Erbas, Mehak Batra

**Affiliations:** 1 School of Psychology and Public Health, La Trobe University, Melbourne, VIC 3086, Australia; y.bekele@latrobe.edu.au (Y.B.); b.erbas@latrobe.edu.au (B.E.); 2 School of Public Health, Bahir Dar University, Bahir Dar 79, Ethiopia

**Keywords:** non-adherence, iron supplementation, urban–rural, sub-Saharan Africa

## Abstract

Background: Adherence to iron supplementation is influenced by systemic barriers, including poor healthcare infrastructure, shortage of healthcare providers, and limited access to antenatal care (ANC) services. These challenges are more pronounced in rural areas. However, evidence on urban–rural disparities in non-adherence to iron supplementation remains limited, particularly in sub-Saharan Africa. This study examined these regional differences, stratified by income levels and national contexts. Method: This analysis utilised Demographic Health Survey (DHS) data conducted between 2015 and 2023 from 26 sub-Saharan African countries, including 287,642 women from urban (*n* = 91,566) and rural areas (*n* = 196,076). The outcome of this study was non-adherence to iron supplementation, defined as taking iron supplementation for less than 90 days during pregnancy. This study examines urban–rural differences in non-adherence stratified by country income levels based on World Bank 2022 income classifications and national context. A chi-square test was used to assess urban–rural differences, with a *p*-value of <0.05 considered statistically significant. Results: Non-adherence was significantly higher in rural areas (68.42%) than in urban areas (51.32%) (*p* < 0.001), with the disparity more pronounced in low-income countries (LICs). Ethiopia, Madagascar, Uganda, and Burundi were among the countries with the highest rural non-adherence, reflecting severe poverty and limited access to ANC. In contrast, Zimbabwe showed an inverse trend, where rural adherence was higher than urban. Conclusions: Rural sub-Saharan Africa has significantly higher non-adherence to iron supplementation, particularly in LICs, likely driven by systemic barriers such as poor infrastructure and limited access to healthcare. This non-adherence in rural areas undermines efforts to improve pregnancy and birth outcomes across the region. Targeted interventions, like those in Zimbabwe, can help address these inequities and improve maternal health outcomes.

## 1. Introduction

Globally, 4.5 million women and infants die annually during pregnancy, childbirth, or the first weeks after birth, with over 95% of these deaths occurring in low- and middle-income countries (LMICs) [1,2,3]. Sub-Saharan Africa bears the highest burden, accounting for 70% of maternal deaths and 41% of newborn deaths, with significant disparities between rural and urban populations [2,3]. Maternal mortality is strongly linked to iron deficiency, a leading cause of maternal anaemia, which increases the risk of haemorrhage, preterm birth, and maternal death, particularly in resource-limited settings [4]. Ensuring early, adequate, and high-quality antenatal care (ANC) services is essential to effectively address these challenges [5].

ANC and immunisation programs are critical platforms for delivering oral iron–folic acid (IFA) [6,7]. However, urban–rural disparities hinder equitable access to these services. In sub-Saharan Africa, ANC coverage stands at 57%, with lower uptake in rural areas (52.19%) compared to urban areas (70.43%) [8]. Similar disparities are evident in immunisation coverage across African countries. For instance, in Ethiopia, urban coverage is 74.3% versus 59.19% in rural areas [9], and in Nigeria, it is 95.5% in urban compared to 75.3% in rural areas [10]. These inequalities are driven by barriers such as geographic isolation, shortages of trained healthcare professionals, limited infrastructure, and financial constraints, which disproportionately affect rural populations and impede access to IFA supplementation and other maternal health interventions [11,12].

Oral IFA supplementation is a key intervention recommended by the World Health Organisation (WHO) to prevent maternal anaemia and improve pregnancy outcomes [6]. In sub-Saharan Africa, women are advised to take IFA supplementation for at least 90 days during pregnancy [13]. However, adherence remains suboptimal across the region, with only 28% of pregnant women meeting the recommended intake [13]. Non-adherence has been associated with an increased risk of maternal anaemia, low birth weight, and neonatal mortality [14,15]. Studies have shown that adherence is particularly poor in LICs, such as the Democratic Republic of the Congo and Burundi, where non-adherence exceeds 91.9% and 97.2%, respectively [11,16]. The problem is even more pronounced in rural areas, where the prevalence of non-adherence was 16% higher compared to urban residents [16]. Systemic challenges such as poor socio-economic status, limited access to healthcare, and logistical challenges are the main contributors to overall non-adherence [11,12,13]. However, existing evidence lacks detailed insights into the extent of urban–rural disparities, particularly when stratified by country income levels and national contexts.

Evidence indicates that poor adherence to iron supplementation is associated with increased odds of adverse pregnancy and birth outcomes, thereby reducing the overall effectiveness of iron supplementation in improving maternal and child health [14,15,17]. For instance, a study conducted in Sri Lanka showed that women who did not adhere to iron supplementation were linked to higher odds of delivering low birth weight compared to those who adhered for at least 90 days [17]. Similarly, studies from African settings further demonstrated that iron supplements for fewer than 90 days do not significantly reduce the odds of low birth weight or post-neonatal mortality compared to non-users [14,15]. The reduced efficacy of iron supplementation due to non-adherence also contributes to ongoing economic burdens associated with preventable adverse maternal and neonatal outcomes [18].

Given the persistently high maternal mortality rates and the critical role of IFA in reducing adverse pregnancy outcomes, this study focuses on urban–rural disparities in IFA non-adherence across 26 Sub-Saharan African countries, categorised into low-income countries (LICs) and lower–middle-income countries (LMICs). Understanding these disparities will provide actionable insights to improve the region’s maternal and newborn health outcomes. Therefore, this study aims to answer the following research questions:

1. What are the differences in non-adherence levels between rural and urban areas in sub-Saharan Africa?
2. How do these levels vary across LICs and LMICs?
3. Which specific countries exhibit the highest levels of non-adherence, and how might this inform the development of targeted interventions?

## 2. Methods

This cross-sectional study analysed data from the Demographic Health Survey (DHS) conducted between 2015 and 2023 across 26 sub-Saharan African countries, including South Africa, Ghana, Burundi, and Nigeria [19]. Specific survey years for each country are listed in Table S1. The DHS, coordinated by the Inner-City Fund (ICF) in collaboration with national governments, collects standardised data every five years on maternal, child, nutrition, reproductive health, and malaria indicators. Ethical approval was granted by the ICF Institutional Review Board (IRB) and the respective IRBs of participating countries. Additional ethical clearance was obtained from La Trobe University under application number HEC23324.

A two-stage cluster sampling technique was used. Enumeration areas (EAs) were selected in the first stage with probability proportional to size. In the second stage, a fixed number of households were systematically selected from each EA. Standardised DHS questionnaires were translated into local languages and administered by trained interviewers under supervision to ensure quality and consistency. This analysis included 287,642 mother–child pairs, of which 68.17% (*n* = 196,076) were from rural areas. Non-adherence to iron supplementation, defined as intake for fewer than 90 days during pregnancy [13], was collected after delivery through maternal recall.

This study was descriptive in nature and aimed at comparing urban–rural differences in non-adherence to iron supplementation across sub-Saharan Africa. No multivariable regression was performed. Instead, chi-square tests assessed group differences, including analyses stratified by country and income classification. While this study did not adjust for confounding variables, we acknowledge that maternal age, education, ANC attendance, and parity may influence adherence and should be explored in future multivariable analyses.

To account for the complex survey design, all analyses applied sampling weights provided by DHS using the svy command in STATA version 18. These weights adjust for clustering, stratification, and unequal selection probabilities, ensuring nationally representative estimates. Country income classifications followed the World Bank’s 2022 categorisation into low-income (LICs) and lower–middle-income countries (LMICs) [15,20]. Statistical significance was set at *p* ≤ 0.05, and all analyses were conducted using STATA version 18 [21].

## 3. Results

Overall, there was a significant disparity in non-adherence levels between rural (68.42%) and urban (51.32%) areas across sub-Saharan Africa (*p* < 0.001), with this urban–rural gap remaining significant across both LICs and LMICs (*p* < 0.05). In LICs, rural areas exhibited significantly higher non-adherence (76.64%) compared to urban areas (58.76%, *p* < 0.001). Five LICs reported rural non-adherence above the LIC regional average, with Ethiopia (rural: 95.63% vs. urban: 89.34%, *p* < 0.001), Madagascar (rural: 82.08% vs. urban: 66.44%, *p* < 0.001), and Uganda (rural: 79.84% vs. urban: 55.92%, *p* < 0.001) showing the highest levels. Burundi had extremely high non-adherence (>95%) in both rural (98.63%) and urban (97.82%) settings, with no significant difference (*p* = 0.714), while Rwanda exhibited slightly lower rural non-adherence (83.70%) than urban (87.06%), but this difference was not significant (*p* = 0.407) (Table 1).

In LMICs, rural non-adherence was below the LICs’ rural regional average (76.64%) in 13 of 15 LMICs and below the sub-Saharan rural average (68.42%) in 10 countries. Five LMICs reported both urban and rural non-adherence levels below 50%, including Senegal (rural: 34.83% vs. urban: 17.65%, *p* < 0.001), Ghana (rural: 41.97% vs. urban: 35.34%, *p* = 0.001), and Kenya (rural: 46.69% vs. urban: 41.14%, *p* < 0.001). South Africa was an exception, where rural non-adherence (36.39%) was slightly lower than urban non-adherence (41.06%), though this difference was not significant (*p* = 0.763). In contrast, Zimbabwe showed the opposite trend, with significantly lower rural non-adherence (54.67%) than urban non-adherence (68.82%, *p* < 0.001) (Table 2).

## 4. Discussion

This study is the first to comprehensively examine urban–rural disparities in iron supplementation non-adherence across sub-Saharan Africa, stratified by country income level and national contexts. Non-adherence was substantially higher in rural areas (68.42%) than in urban areas (51.32%, *p* < 0.001), with the gap more pronounced in LICs (rural: 76.64% vs. urban: 58.76%, *p* < 0.001) than in LMICs (rural: 59.33% vs. urban: 48.14%, *p* < 0.001). An exception was observed in Zimbabwe, where rural non-adherence was significantly lower than urban non-adherence.

Low adherence to iron supplementation reduces the effectiveness of interventions aimed at improving pregnancy and birth outcomes [6]. Poor adherence has been associated with increased risks of maternal anaemia, low birth weight, and neonatal mortality [17]. Evidence suggests that iron intake below the recommended 90-day threshold during pregnancy is insufficient to mitigate these adverse outcomes [14,15]. Moreover, the reduced effectiveness of supplementation contributed to substantial economic losses. In sub-Saharan Africa, for instance, the economic burden associated with low birth weight is estimated to be equivalent to approximately 10% of national per capita income [18]. These findings underscore the critical need to address barriers to adherence, particularly in rural areas, to improve maternal and neonatal health outcomes across the region.

### 4.1. Urban–Rural Disparities in LICs

Four LICs, including Ethiopia, Madagascar, and Uganda, reported rural non-adherence levels that exceeded the regional rural average of 76.64%. These elevated levels are likely driven by systemic barriers such as widespread poverty, limited access to healthcare, and low antenatal care (ANC) utilisation. For example, the rural poverty rate in Uganda is 55%, and in Madagascar, it is 79.9%, significantly higher than the rural average in sub-Saharan Africa of 17.2% [22,23,24]. In addition, ANC coverage remains below 50% in Ethiopia and Madagascar, limiting opportunities for pregnant women to access iron supplementation [25,26].

Burundi presents an extreme case, with non-adherence rates exceeding 95% in both rural and urban settings. This may stem from chronic underfunding, severe healthcare workforce shortages, and the long-term impact of the country’s 13-year civil war [27,28]. The lack of a significant rural–urban difference suggests that barriers to iron supplementation exist nationwide, requiring structural reforms and increased investment in maternal health services.

### 4.2. Trends in LMICs and Promising Policy Interventions

In contrast, LMICs show relatively lower rural non-adherence rates, with 13 countries reporting rates below the LIC rural average of 76.64% and five countries—including Senegal, Ghana, and Kenya—reporting non-adherence levels below 50%. This may reflect higher national healthcare expenditures closely tied to economic growth and improved healthcare access [29]. Evidence suggests that stronger health financing models in LMICs contribute to better maternal health outcomes [30,31].

In Zambia, non-adherence levels were among the lowest (rural: 20.61%, urban: 19.55%). This may be attributed to the national implementation of Safe Motherhood Action Groups (SMAGs), community-based initiatives designed to improve maternal and newborn health by addressing delays in health-seeking and engaging communities [31,32]. SMAGs have been linked to increased ANC attendance and enhanced maternal health awareness, especially in rural areas [33]. In Zimbabwe, the Results-Based Financing (RBF) program has been key in reducing financial barriers and improving rural regions’ access to maternal health services [30,34,35]. RBF provides financial incentives to healthcare facilities based on measurable improvements in service delivery, particularly maternal and child health outcomes [36,37]. Zimbabwe’s high female literacy rate (99%), substantial ANC coverage (71.7% receiving at least four visits), and ongoing rural health infrastructure development have also contributed to improved adherence [38,39]. These examples illustrate how well-designed national programs and policy measures can improve iron supplementation adherence and maternal health outcomes in resource-limited settings.

### 4.3. Policy Implications and Future Directions

Findings from this study highlight the urgent need for targeted strategies to improve IFA adherence in rural sub-Saharan Africa, particularly in LICs. Addressing barriers such as healthcare access gaps, workforce shortages, and socio-economic disparities will be critical. Countries with high non-adherence, such as Ethiopia and Burundi, may benefit from scaling up proven interventions like community-based maternal health education to increase awareness and increase investments to strengthen rural health services.

Implementing targeted outreach programs, particularly a community-based approach involving community health workers (CHWs), can significantly enhance maternal health intervention uptake. CHWs are strategically positioned to identify and engage pregnant women by leveraging integrated service platforms, such as vaccination and CHW service packages. Additionally, introducing or strengthening cash transfer (CT) programs for women experiencing financial hardship is crucial. CTs have demonstrated effectiveness in addressing key social determinants of health by reducing economic barriers and improving access to essential maternal health services [40]. Expanding these platforms can substantially improve the uptake of IFA supplementation. Future research, particularly qualitative or mixed-method research, is also needed to explore cultural beliefs and systemic barriers influencing adherence behaviours and inform the development of culturally sensitive interventions.

The strength of this study lies in its large sample size (*n* = 287,642) and detailed analysis of urban–rural disparities, stratified by country income level and national contexts. However, this study has several limitations. First, mothers’ self-reported adherence to iron supplementation after delivery may be subject to recall bias, especially when reporting events from months or years earlier. Second, as a secondary analysis of DHS data, this study was constrained by the variables available and could not assess key factors such as the quality of counselling or the influence of healthcare providers. Differences in survey years across countries may also introduce temporal variation. While categorising countries by income level (LICs vs. LMICs) provides a useful comparative framework, it may mask significant within-country differences, such as disparities between urban slums and rural communities. Therefore, findings should be interpreted cautiously, and future research should consider more granular analyses to account for subnational heterogeneity. This was a descriptive, cross-sectional study focused on highlighting urban–rural disparities rather than establishing associations or causality. The explanatory depth is limited by the absence of multivariate analysis adjusting for confounders such as maternal age, education, healthcare access, and ANC visits. Moreover, systemic barriers, including structural and contextual factors like inadequate health infrastructure, workforce shortages, poverty, low health literacy, supply chain disruptions, distance to clinics, and cultural beliefs, were not directly assessed in this study. Therefore, future quantitative research that accounts for potential confounding factors to estimate effect sizes, alongside qualitative research to explore the underlying complexities, is essential to inform effective and context-specific interventions.

In conclusion, rural non-adherence to iron supplementation remains a significant challenge in sub-Saharan Africa, particularly in LICs. The extreme non-adherence levels in Burundi highlight the urgency of addressing long-standing systemic barriers, including inadequate healthcare budgeting and shortages of healthcare providers. However, promising trends in Zimbabwe and Zambia demonstrate the potential of targeted, evidence-based interventions. To address these disparities, potential interventions should include expanding access to ANC services in rural settings, strengthening community-based health education, and reducing financial and geographic barriers to care. Examples include conditional cash transfers, transportation vouchers, and mobile health outreach. The Results-Based Financing program in Zimbabwe demonstrates how targeted, performance-linked funding can improve maternal health service delivery and support more equitable access to supplementation. Future research should explore the complex interplay of socio-cultural, economic, healthcare, and policy factors to inform sustainable, equity-focused solutions for improving adherence in resource-limited settings.

## Figures and Tables

**Table 1:** **Country-specific** urban–rural differences in non-adherence to iron supplementation in low-income countries in sub-Saharan Africa (weighted sample).

Countries	Non-Adherence to Iron Supplementation		
	Rural Areas	Urban Areas	*p*-Value
Burkina Faso	1998 (46.11)	474 (33.04)	<0.001
Burundi	7917 (98.63)	849 (97.82)	0.714
Ethiopia	6219 (95.63)	836 (89.34)	<0.001
Gambia	620 (39.44)	1135 (37.20)	0.037
Liberia	780 (52.22)	955 (49.93)	0.002
Madagascar	6041 (82.08)	965 (66.44)	<0.001
Malawi	7605 (67.33)	1112 (58.67)	<0.001
Mali	3176 (70.73)	543 (52.51)	<0.001
Rwanda	4311 (83.7)	966 (87.06)	0.407
Sierra Leon	2319 (64.96)	1120 (60.24)	0.001
Uganda	6010 (79.84)	1475 (67.16)	<0.001
Total	46,994 (76.64)	10,431 (58.76)	<0.001

Footnote: *p*-value derived from the chi-square test.

**Table 2:** **Country-specific** urban and rural differences in non-adherence to iron supplementation in lower- and middle-income countries in sub-Saharan Africa (weighted sample).

Countries	Non-Adherence to Iron Supplementation		
	Rural Areas	Urban Areas	*p*-Value
Angola	2230 (80.76)	2586 (55.92)	<0.001
Benin	2396 (51.12)	1084 (38.00)	<0.001
Cameroon	1957 (59.37)	975 (34.42)	<0.001
Cote d’Ivoire	1873 (73.95)	1734 (67.94)	<0.001
Gabon	166 (62.28)	1224 (45.94)	<0.001
Ghana	1019 (41.97)	789 (35.34)	0.001
Guinea	2977 (79.65)	981 (65.59)	<0.001
Kenya	2743 (46.69)	1401 (40.14)	<0.001
Mauritania	2061 (70.74)	1445 (61.08)	<0.001
Nigeria	9283 (73.97)	4501 (57.34)	<0.001
Senegal	779 (34.83)	214 (17.65)	<0.001
South Africa	323 (36.39)	673 (41.06)	0.763
Tanzania	2542 (61.13)	816 (50.62)	<0.001
Zambia	811 (20.61)	501 (19.55)	0.748
Zimbabwe	1771 (54.67)	1082 (68.82)	<0.001
Total	32,931 (59.33)	20,005 (48.14)	<0.001

Footnote: *p*-value derived from the chi-square test.

## Data Availability

Data are available upon request on The DHS program website at https://www.dhsprogram.com/data/available-datasets.cfm, accessed on 22 February 2024.

## Supplementary Materials

The following supporting information can be downloaded at https://www.mdpi.com/article/10.3390/ijerph22060964/s1, Table S1: List of included sub-Saharan African countries and survey years.
