# Supplementary material for: Urban–Rural Disparities in Non-Adherence to Iron Supplementation Among Pregnant Women Aged 15 to 49 in Sub-Saharan Africa
Source: Int J Environ Res Public Health. 2025 Jun 19;22(6):964. doi: 10.3390/ijerph22060964 (PMC12192883; doi:10.3390/ijerph22060964)
Supplement: Supplementary file 1 [file ijerph-22-00964-s001.zip › ijerph-3610857-supplementary.pdf]

Table S1: List of included sub-Saharan African countries and survey years		
**Countries**	**Country income level**	**Survey Year**
Angola	LMICs	2015/16
Benin	LMICs	2017/18
Burkina Faso	LICs	2021
Burundi	LICs	2016/17
Cameroon	LMICs	2018
Cote d'Ivoire	LMICs	2021
Ethiopia	LICs	2016
Gabon	LMICs	2019/20
Gambia	LICs	2019/20
Ghana	LMICs	2022
Guinea	LMICs	2018
Kenya	LMICs	2022
Liberia	LICs	2019/20
Madagascar	LICs	2021
Malawi	LICs	2015/16
Mali	LICs	2018
Mauritania	LMICs	2019-21
Nigeria	LMICs	2018
Rwanda	LICs	2019/20
Senegal	LMICs	2023
Sierra Leon	LICs	2019
South Africa	LMICs	2016
Tanzania	LMICs	2022
Uganda	LICs	2016
Zambia	LMICs	2018
Zimbabwe	LMICs	2015
